# Proteomic Assessment of C57BL/6 Hippocampi after Non-Selective Pharmacological Inhibition of Nitric Oxide Synthase Activity: Implications of Seizure-like Neuronal Hyperexcitability Followed by Tauopathy

**DOI:** 10.3390/biomedicines10081772

**Published:** 2022-07-22

**Authors:** Jhana O. Hendrickx, Charlotte Adams, Anne Sieben, Kris Laukens, Debby Van Dam, Guido R. Y. De Meyer

**Affiliations:** 1 Laboratory of Physiopharmacology, University of Antwerp, 2610 Antwerp, Belgium; jhana.hendrickx@uantwerpen.be; 2 Department of Mathematics and Computer Science, University of Antwerp, 2610 Antwerp, Belgium; charlotte.adams@uantwerpen.be (C.A.); kris.laukens@uantwerpen.be (K.L.); 3 Biomedical Informatics Network Antwerpen (Biomina), University of Antwerp, 2020 Antwerp, Belgium; 4 Laboratory of Neurochemistry and Behaviour, Experimental Neurobiology Unit, 2610 Antwerp, Belgium; anne.sieben@uantwerpen.be (A.S.); debby.vandam@uantwerpen.be (D.V.D.); 5 Department of Neurology and Alzheimer Center, University of Groningen, 9713 GZ Groningen, The Netherlands; 6 University Medical Center Groningen, 9700 RB Groningen, The Netherlands

**Keywords:** hippocampus, L-NAME, nitric oxide, proteomics, hyperexcitability, ribosomal dysfunction, tauopathy, mitochondrial dysfunction

## Abstract

Nitric oxide (NO) is a small gaseous signaling molecule responsible for maintaining homeostasis in a myriad of tissues and molecular pathways in neurology and the cardiovasculature. In recent years, there has been increasing interest in the potential interaction between arterial stiffness (AS), an independent cardiovascular risk factor, and neurodegenerative syndromes given increasingly epidemiological study reports. For this reason, we previously investigated the mechanistic convergence between AS and neurodegeneration via the progressive non-selective inhibition of all nitric oxide synthase (NOS) isoforms with N(G)-nitro-L-arginine methyl ester (L-NAME) in C57BL/6 mice. Our previous results showed progressively increased AS *in vivo* and impaired visuospatial learning and memory in L-NAME-treated C57BL/6 mice. In the current study, we sought to further investigate the progressive molecular signatures in hippocampal tissue via LC–MS/MS proteomic analysis. Our data implicate mitochondrial dysfunction due to progressive L-NAME treatment. Two weeks of L-NAME treatment implicates altered G-protein-coupled-receptor signaling in the nerve synapse and associated presence of seizures and altered emotional behavior. Furthermore, molecular signatures implicate the cerebral presence of seizure-related hyperexcitability after short-term (8 weeks) treatment followed by ribosomal dysfunction and tauopathy after long-term (16 weeks) treatment.

## 1. Introduction

Although a small gaseous molecule in the human body, nitric oxide (NO) is one of the most important signaling molecules and neurotransmitters in the central and peripheral nervous system [1,2]. NO is produced by NOS through the enzymatic conversion of L-arginine to citrulline. Three main NOS isoforms exist: inducible NOS (iNOS), endothelial NOS (eNOS), and neuronal NOS (nNOS). The functionality of these enzymes depends on their localization of action: iNOS is expressed in the cytosol of glial cells upon brain injury or inflammation, eNOS is constitutively active and expressed in endothelial cells in the membrane-bound state, while nNOS is constitutively active and expressed in neuronal cytosol [3,4].

Despite their functionality in the nervous system, these NOS isoforms also maintain cardiovascular homeostasis, including the protection of the vessel from injurious consequences and the modulation of vascular dilator tone. Until recently, eNOS was considered the most prominent NOS isoform in the cardiovascular system given its main expression in endothelial cells regulating basal and dynamic blood vessel diameter changes [5,6]. However, a previous study on human explanted left ventricular heart tissue exemplified the importance of nNOS, rather than eNOS and iNOS, in the pathophysiology of ischemic heart disease [7] Additionally, nNOS-deficient mice were shown to develop pathological left ventricular remodeling and functional decline after myocardial infarction [8,9]. In addition, iNOS is expressed in most cardiovascular tissues, e.g., endothelial cells, vascular smooth muscle cells, and cardiomyocytes, as a host–cell response upon exposure to cytokines or lipopolysaccharides [10].

NO is considered a double-edged sword, however. In normal physiological conditions, it exerts an anti-inflammatory function [11,12], while in cardiovascular pathology, i.e., arterial stiffness (AS) [13,14] and in neurodegenerative diseases [15,16], NO is also considered a pro-inflammatory mediator. Recently, there has been a growing interest in the potential mechanistic convergence between AS and neurodegenerative syndromes since epidemiological studies increasingly report an independent interplay between both pathologies [17,18]. In essence, AS relates the pulsatile blood flow from the heart to the brain. Given its extensive microvasculature, the brain is not only a high-flow, but also a low-resistant organ that is continuously exposed to cardiac pulsatile pressures and mechanical forces [19]. There is a consensus that an increased pulse wave velocity (PWV), an *in vivo* parameter of AS, is linked to deteriorating psychomotor speed, semantic fluency and verbal learning, and a faster cognitive decline [17,20].

Previously, we sought to investigate the effect of AS on the progression of spontaneous neurodegeneration in a non-selective, pharmacological NOS dysfunction mouse model of AS. More specifically, 0.5 mg/mL N(G)-nitro-L-arginine methyl ester (L-NAME) was added to the drinking water of C57BL/6 mice for 2, 8, and 16 weeks from the age of 8 weeks onward. As expected, we measured significantly increased PVW values in L-NAME-treated compared to non-treated C57BL/6 mice after 2, 8, and 16 weeks (4.4 ± 0.4 ** vs. 2.8 ± 0.2, 4.5 ± 0.3 ** vs. 3.4 ± 0.2, 5.5 ± 0.4 *** vs. 3.4 ± 0.2 with ** *p* < 0.01 and *** *p* < 0.001) which was progressively significant over time (factorial ANOVA, *p* = 0.014). Moreover, we found that this long-term non-selective inhibition of all NOS isoform activity with L-NAME led to deteriorated hippocampal-dependent visuospatial learning and memory in C57BL/6 mice as assessed with a Morris water maze test. This outcome therefore highlights the potential contribution of NOS activity in the convergence between AS and cognitive decline [21]. In the present study, we aimed to further investigate the progressive generation of molecular signatures in degenerating hippocampal tissue of previously characterized L-NAME-treated C57Bl/6 mice [21] via LC–MS/MS proteomic analysis.

## 2. Materials and Methods

### 2.1. Experimental Animals and Tissue Collection

The pharmacological, non-selective NOS inhibition was induced by adding 0.5 mg/mL N(G)-nitro-L-arginine methyl ester (L-NAME, Sigma-Aldrich^®^ Solutions, Hoeilaart, Belgium) to the drinking water of male C57BL/6 mice (The Jackson Laboratory, Bar Harbor, ME, USA) from the age of 8 weeks onward for 2 weeks (control *n* = 9; treated *n* = 10), 8 weeks (control *n* = 11; treated *n* = 11), or 16 weeks (control *n* = 10; treated *n* = 7). Compared to previous studies [22,23], a lower L-NAME dosage was administered in order to obtain only partial eNOS activity inhibition in the aortic vessel wall. All mice were socially housed up to a maximum of eight animals in standard mouse cages under conventional laboratory conditions, with a constant room temperature (22 ± 2 °C) and humidity (55 ± 5%), and an artificial day/night cycle of 12 h/12 h (lights on at 8:00 a.m.). Food and water were provided ad libitum. The average L-NAME intake was assessed by weighing the drinking bottle at the beginning and end of each week per animal cage, respectively, after which the L-NAME drinking water was refreshed. The average L-NAME intake was 3.3, 2.9, and 3.3 mg/day per treated animal at 2, 8, and 16 weeks of treatment, respectively. Experiments were approved by the Animal Ethics Committee of the University of Antwerp (ECD approval No. 2017/53, approved on 26 July 2017) and were carried out in accordance with the U.K. Animals (Scientific Procedures) Act, 1986 and associated guidelines, and EU Directive 2010/63/EU on the protection of animals used for scientific purposes. This manuscript was prepared in accordance with the *Animal Research: Reporting of In Vivo Experiments (ARRIVE) Guidelines* [24]. After a 2-week experimental battery as previously reported [21], mice were humanely killed by perforation of the diaphragm while under deep anesthesia (sodium pentobarbital, Sanofi, Diegem, Belgium), 250 mg/kg, i.p. [25]). Subsequently, the hippocampus of one cerebral hemisphere (five mice per treatment group at 2, 8 and 16 weeks of treatment, 60 samples in total) were collected and immediately frozen in liquid nitrogen.

### 2.2. Sample Preparation

Hippocampal tissues were homogenized in 2 mL urea lysis buffer containing 8 M urea and 20 mM HEPES pH 8.0. Next, the homogenized samples were sonicated using a 3 mm probe with 3 pulses of 15 s at an amplitude of 20%, with incubation on ice for 1 min between pulses. In order to remove insoluble components, samples were centrifugated for 15 min at 20,000× *g* at room temperature. Next, proteins were reduced by the addition of 5 mM dithiothreitol and incubation for 30 min at 55 °C and then alkylated by the addition of 10 mM iodoacetamide and incubation for 15 min at room temperature in the dark. The Bradford assay (Bio-Rad, Hercules, CA, USA) was executed to measure the protein concentration, and a total of 100 µg protein was used from each sample to continue the protocol. Samples were further diluted with 20 mM HEPES pH 8.0 to a final urea concentration of 4 M. Afterwards, proteins were digested with 1 µg LysC (FUJIFILM Wako Chemicals USA Corporation, Richmond, VA, USA) (1/100, *w*/*w*) for 4 h at 37 °C, whereafter samples were again diluted to 2 M urea and digested with 1 µg trypsin (Promega Corporation, Madison, WI, USA) (1/100, *w*/*w*) overnight at 37 °C. The resulting peptide mixture was acidified by the addition of 1% trifluoroacetic acid (TFA). After 15 min incubation on ice, samples were centrifuged for 15 min at 1780× *g* at room temperature to remove insoluble components. Next, peptides were purified on SampliQ SPE C18 cartridges (Agilent Technologies, Santa Clara, CA, USA), whereby columns were first washed with 1 mL 100% acetonitrile (I) and pre-equilibrated with 3 mL of solvent A (0.1% TFA in water/I (98:2, *v/v*)), before samples were loaded on the column. After peptide binding, the column was rewashed with 2 mL of solvent A, and peptides were eluted twice with 750 µL elution buffer (0.1% TFA in water/I (40:60, *v/v*)). Afterwards, purified peptides were vacuum dried in HPLC inserts and stored at −20 °C until LC–MS/MS analysis.

### 2.3. LC–MS/MS Analysis

Purified peptides were redissolved in 20 µL solvent A. A volume of 2 µL per sample was injected for LC–MS/MS analysis on an Ultimate 3000 RSLCnano system (Thermo Fisher Scientific, Waltham, MA, USA) in line connected to a Q Exactive HF Biopharma mass spectrometer (Thermo Fisher Scientific, Waltham, MA, USA). Trapping was performed at 10 μL/min for 4 min in loading solvent A on a 20 mm trapping column (developed in-house, 100 μm internal diameter (I.D.), 5 μm beads, C18 Reprosil-HD, Dr. Maisch, Ammerbuch-Entringen, Germany). The peptides were separated on a nanoEase column (MZ HSS T3 1.8 μm, 75 μm × 250 mm, part: 186008818, Waters, Milford, MA, USA) at a constant temperature of 50 °C. Next, peptides were eluted by a non-linear increase from 1 to 55% MS solvent B (0.1% FA in water/I (2:8, *v*/*v*)) over 100 min, at a flow rate of 300 nL/min, followed by a 5 min wash reaching 97% MS solvent B and re-equilibration with 99% MS solvent A (0.1% FA in water). The Q Exactive HF Biopharma mass spectrometer (Thermo Fisher Scientific, Waltham, MA, USA) was operated in data-dependent mode, automatically switching between MS and MS/MS acquisition for the 16 most abundant ion peaks per MS spectrum. Full-scan MS spectra (375–1500 *m*/*z*) were acquired at a resolution of 60,000 in the Orbitrap analyzer (Thermo Fisher Scientific, Waltham, MA, USA) after accumulation to a target value of 3,000,000. The 16 most intense ions above a threshold value of 13,000 (minimum AGC of 1000) were isolated for fragmentation at a 28% normalized collision energy. The C-trap was filled at a target value of 100,000 for a maximum of 80 ms. The MS/MS spectra (200–2000 *m*/*z*) were acquired at a resolution of 15,000 in the Orbitrap analyzer with a fixed first mass of 145 *m*/*z*. Only peptides with charge states ranging from +2 to +6 were included for fragmentation, and the dynamic exclusion was set to 12 s. Qcloud was used to control instrument longitudinal performance during the project [26].

### 2.4. Data Analysis and Retrieval of Differentially Expressed Proteins

Using the Andromeda search engine from MaxQuant (Version 1.6.11.0), all acquired proteomic data were searched against the mouse proteome acquired from the Swiss-Prot Reference Proteome database (database release version of June 2019 containing 22,282 mouse protein sequences [27]). Default settings were used, including a false discovery rate (FDR) set at 1% on peptide spectrum matches (PSM) and peptide level, a fragment ion mass tolerance of 20 ppm, and a precursor mass tolerance of 4.5. Only proteins with at least one unique or razor peptide with a minimum length of 7 amino acids were retained leading to the identification of 3222 proteins.

Enzyme specificity was set as C-terminal to arginine and lysine, also allowing cleavage at proline bonds with a maximum of two missed cleavages. Variable peptide modifications were set to acetylation of protein N-termini and oxidation of methionine residues, while carbamidomethylation of cysteine residues was set as fixed modification. The identified peptides were further analyzed with a robust regression model using the MSqRob2 R package [28,29,30] to generate a list of differentially expressed proteins (DEPs). Peptide intensities were Log2-transformed, filtered, normalized, and finally summarized into protein expression values with robust model-based summarization. Proteins were considered as differentially expressed if the Bonferroni–Hochberg PAdj was lower than 0.05 and the Log2 fold change was higher than 1. For this experiment the ‘Age × Treatment’ model was applied with additional inferences per treatment duration.

### 2.5. Gene Ontology and Pathway Analysis

The DEPs of the acquired datasets were functionally annotated using gene ontology and pathway analysis. Gene ontology analysis of biological process, molecular function and cellular component, as well as pathway analysis using the Kyoto Encyclopedia of Genes (KEGG) [27,28], The Reactome Knowledgebase (REACTOME) [29], and WikiPathways [30] databases, was performed by importing the DEP lists in the web-based g:Profiler database [31] on a *Mus musculus* (Mouse) background. Query outputs were visualized using GraphPad Prism (version 9.1.2 for Windows, GraphPad Software, San Diego, CA, USA) and Cytoscape software [32], respectively. Mouse phenotypes related to DEP lists were obtained via the Mouse Genomic Informatics (MGI) Mammalian Phenotype Level 4 (2021) database in the web-based gene lists enrichment analysis tool, Enrichr [33].

### 2.6. Histological Analysis

Cerebral tissues were immediately fixed in 4% formaldehyde (BDH Prolabo, VWR Chemicals, Radnor, PA, USA) for 24 h, whereafter they were dehydrated overnight in 60% isopropanol (BDH Prolabo, VWR Chemicals, Radnor, PA, USA) and embedded in paraffin. Next, serial cross sections (5 µm) of tissues were prepared for histological analysis. All images were acquired using the Universal Grab 6.1 software with an Olympus BX40 microscope. The presence of tauopathy features, i.e., neurofibrillary tangles (NFT) and neuronal intracytoplasmic inclusions (NCI), were comprehensively scored by a trained neuropathologist on pTAU-stained (Thermo Fisher Scientific, Waltham, MA, USA, # MN1020, 1:3000 dilution) sagittal brain slices of the hippocampus and cortex. The presence or absence of NFT and NCI on sagittal brain slices was annotated as ‘1’ or ‘0’ for each animal.

## 3. Results

### 3.1. Mitochondrial Functionality Is Progressively Affected by L-NAME

After analyzing individual treatment durations, the overall effect of treatment and treatment duration was further examined through a two-way ANOVA. In total, three DEPs were retrieved (Table 1). Firstly, we found the Timm10 protein, a mitochondrial intermembrane chaperone that participates in the import and insertion of multi-pass transmembrane proteins into the mitochondrial inner membrane. Secondly, we obtained the Tmsb10 protein, a cytoskeletal protein that binds to and sequesters actin monomers and therefore inhibits actin polymerization. Lastly, we found the Lsamp protein, a GPI-anchored cell adhesion molecule heavily expressed in limbic and limbic-associated regions of the developing and adult brain that mediates selective neuronal growth and axon targeting [34,35].

### 3.2. Two Weeks of L-NAME Treatment Implicates Altered G-Protein-Coupled-Receptor Signaling in the Nerve Synapse and Associated Presence of Seizures and Altered Emotional Behavior

In total, 9 DEPs were retrieved between the 2-week L-NAME-treated and -untreated protein datasets (Table 2) existing of a wide variety of protein classes, i.e., cytoskeletal protein, metabolite conversion enzyme, nucleic acid metabolism protein, protein modifying enzyme, translational protein, and transmembrane signal receptor.

**Figure 1:**
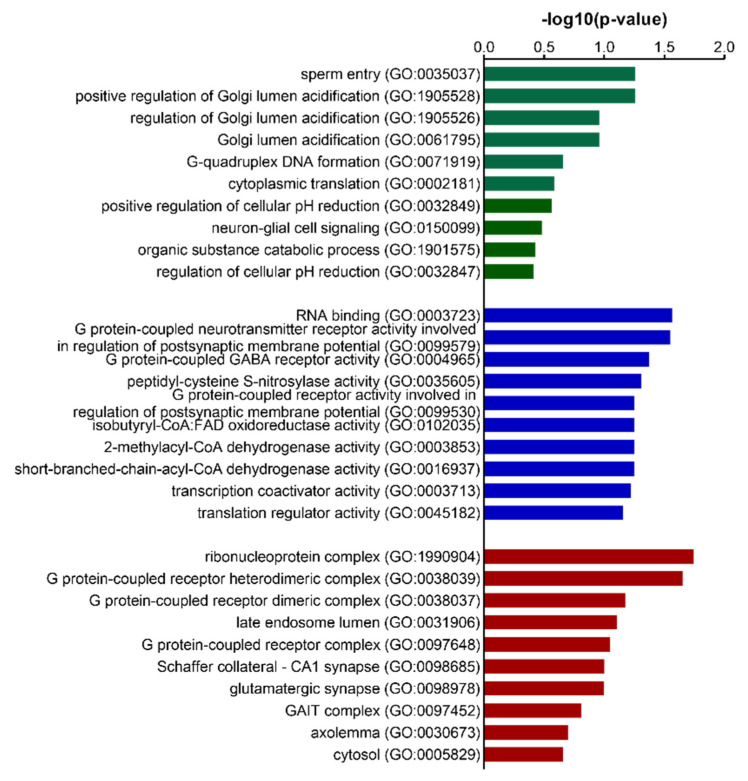
Gene Ontology analysis of the DEP list of 2-week L-NAME-untreated and -treated hippocampal tissue of C57BL/6 mice. Top 10 most significant GO hits for biological process (green bars), molecular function (blue bars), and cellular component (red bars). The *y*-axis depicts GO terms with their associated GO-ID, and the *x*-axis the associated −log10 (*p*-value).

Gene ontology analysis of DEPs (Table 2) resulted in indications of Golgi lumen acidification as biological process (Figure 1), RNA binding and synaptic G-protein-coupled neurotransmitter and GABA receptor activity as molecular functions (Figure 1), whereby proteins strongly belonged to ribonucleoprotein and G-protein-coupled receptor complexes (Figure 1). Pathway analysis of the DEP list (Table 2) did not result in enriched pathways.

Then the mouse mammalian phenotypes associated with the DEP list (Table 2) were retrieved from the MGI Mammalian Phenotype Level 4 2021 database. We found that our dataset was enriched, among others, for phenotypes associated with absence/audiogenic seizures and abnormal or affected emotional behavior (Table 3).

### 3.3. Eight Weeks of L-NAME Treatment Is Associated with Cerebral Actin Depolymerization

Only one DEP (Table 4), Tmsb10, was retrieved between the 8-week L-NAME-treated and untreated protein datasets. Tmsb10 is a cytoskeletal protein that binds to and sequesters actin monomers and therefore inhibits actin polymerization [34].

### 3.4. Sixteen Weeks of L-NAME Treatment Is Associated with Ribosomal Dysfunction and Tauopathy

A list of DEPs (Appendix A) was compiled by the robust summarization method from the msqrob2 R package [36,37,38]. Proteins were considered as differentially expressed if the Bonferroni–Hochberg PAdj-value was lower than 0.05, and the fold change was higher than 1.5. The DEP list (Table 4) was further used as the input for the creation of graphs.

**Figure 2:**
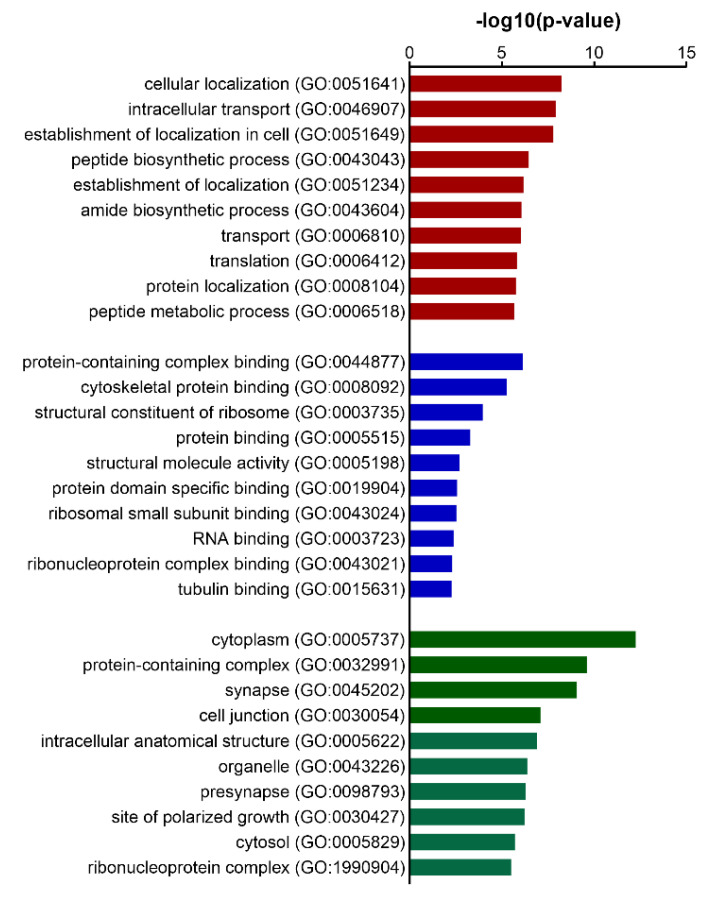
Gene Ontology analysis of the DEP list of 16-week L-NAME-untreated and -treated hippocampal tissue of C57BL/6 mice. Top 10 most significant GO hits for biological process (green bars), molecular function (blue bars), and cellular component (red bars). The *y*-axis depicts GO terms with their associated GO-ID, and the *x*-axis the associated −log10 (*p*-value).

In total, 120 DEPs (Appendix A) were retrieved between the 16-week L-NAME-untreated and -treated protein datasets comprising a wide variety of protein classes, i.e., cytoskeletal proteins, membrane traffic proteins, metabolite conversion enzymes, nucleic acid metabolism proteins, and translational proteins, among others. The most significantly altered protein was MAPT, microtubule-associated protein tau, a key protein involved in neurodegenerative diseases such as AD and tauopathies [34,39]. Gene ontology analysis of DEPs (Appendix A) resulted in indications of intracellular localization and transport as biological process (Figure 2), and cytoskeletal and ribosomal protein binding as molecular functions (Figure 2), whereby proteins were strongly situated in the cytoplasm, synapse, and in protein complexes (Figure 2).

**Figure 3:**
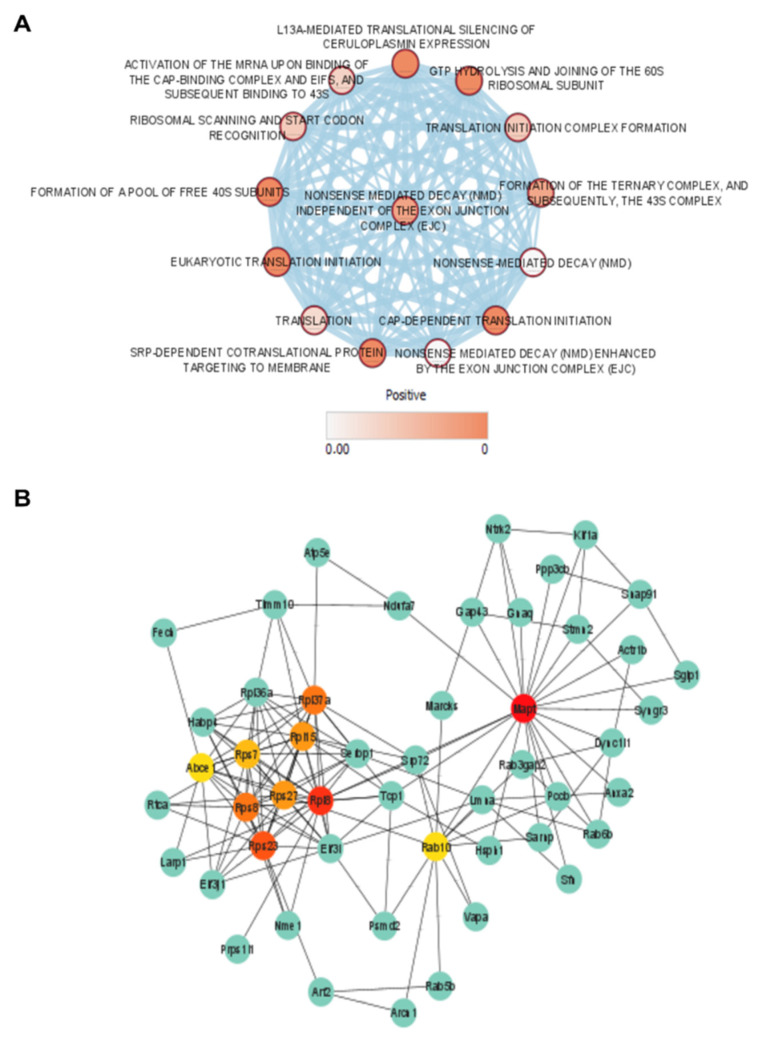
Network and pathway enrichment analysis of the DEP list of 16-week L-NAME-untreated and -treated hippocampal tissue of C57BL/6 mice. (**A**) Pathway enrichment analysis output from g:Profiler including the KEGG, REACTOME, and WikiPathways databases visualized via the Cytoscape Enrichment Map. Each circle depicts individual gene sets ranked from low (white) to high (red) Q-value positivity. Interactions between different gene sets are indicated by blue lines. (**B**) Protein network (Cytoscape) from the String output (version 11.5, available on: https://string-db.org (accessed on 25 January 2022)) of the complete DEP list. The protein network only depicts proteins displaying a node interaction with top 10 essential nodes (cytoHUBBA in Cytoscape) ranked by node degree from highly essential (red) to normal (green).

Pathway enrichment analysis of the DEP list (Table 4) revealed a strong clustering of pathways involved in RNA surveillance (i.e., nonsense-mediated decay (nmd) independent of the exon junction complex (ejc)) and ribosomal translation (i.e., CAP-dependent translation initiation, eukaryotic translation initiation, GTP hydrolysis, and joining of the 60S ribosomal subunit) (Figure 3A).

A protein network was generated whereby only proteins with a node degree were included. Next, the top 10 essential protein hubs were calculated based on their node degree (Figure 3B). We found three highly essential protein hubs, i.e., Rpl8, Rps23, and Mapt. The Rpl8 and Rps23 proteins, 60S and 40S ribosomal proteins, respectively, were clustered together in a network with other essential hubs related to the ribosomal protein machinery (Rpl37a, Rpl15, Rps7, Rps27, Rps8). Mapt, microtubule-associated protein tau, was solely situated, although it could be linked to the Rpl8–Rps23 hub network via the Rab10 hub protein. Rab10 (Ras-related protein Rab-10) is a small GTPase that serves as a key regulator of intracellular membrane trafficking, mediating the formation, transport, and fusion of vesicles with membranes [40].

Next, mouse mammalian phenotypes associated with the DEP list (Appendix A) were retrieved from the MGI Mammalian Phenotype Level 4 2021 database. We found that our dataset was enriched, among others, for phenotypes associated with abnormal synaptic vesicle number, abnormal axon extension and abnormal miniature excitatory postsynaptic currents (Table 5).

### 3.5. Histopathological Analysis Reveals an Increased Presence of Neuronal Intracytoplasmic Inclusions after Sixteen Weeks of L-NAME Treatment

**Figure 4:**
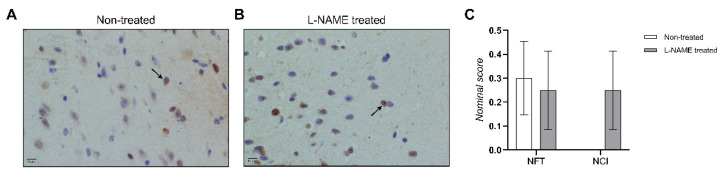
Histopathological analysis of pTAU stained cerebral tissue of one hemisphere of non-treated and 16-weeks L-NAME-treated mice. Representative images of (**A**) non-treated and (**B**) L-NAME-treated of hippocampal cortex tissue, respectively. There were sparse neuronal intracytoplasmic inclusions (NCI) (black arrows), which were considered pretangles or NCI. In none of the cases was there a fulminant neurofibrillary tangle pathology. (**C**) Neuropathological scoring of hippocampal and cortical tissue of one hemisphere of 16-week control and L-NAME-treated animals. NFT = neurofibrillary tangles, and NCI = neuronal intracytoplasmic inclusions. Data are presented as mean ± SEM. Scale bar = 10 µm.

As a result of the molecular findings in hippocampal tissue of 16-week L-NAME-treated animals, immunohistological staining of the pTAU protein was performed on hippocampal and cortical tissue of 16-week (non)-treated animals and comprehensively scored by a neuropathologist. We found an equal presence of NFT in both non-treated and L-NAME-treated cerebral tissue (Figure 4). Additionally, we only observed neuronal intracytoplasmic inclusions in L-NAME-treated cerebral tissue (Figure 4).

## 4. Discussion

In previous work we studied the effect of AS on the progression of neurodegeneration in C57BL/6 mice. More specifically, non-selective NOS inhibition was induced by pharmacological treatment of male C57BL/6 mice from the age of 8 weeks onward with L-NAME for a period of 2, 8, and 16 weeks. As expected, we found significantly increased PWV values in L-NAME-treated animals compared to controls after 2, 8, and 16 weeks of treatment, which was progressively significant over time. Moreover, we found that long-term L-NAME treatment led to impaired visuospatial learning and memory [21]. The goal of this study was to further explore the observed progressive cognitive decline because of L-NAME treatment via proteomic analysis of hippocampal tissue.

We started the proteomics analysis by studying the progressive effect of L-NAME on hippocampal tissue of (un)treated C57BL/6 mice. We were able to find three DEPs: Timm10, Tmsb10, and Lsamp. Tmsb10 is an actin-related cytoskeletal protein important for the orchestration of the structural organization of synapses and thus their formation, stability, and plasticity [34]. Additionally, we obtained the mitochondrial inner membrane chaperone protein, Timm10. This outcome refers to the well-known importance of NO in the mitochondrial inner membrane of cells, to which NO binds to inhibit members of the electron transport chain, i.e., complex III and cytochrome c oxidase, hereby controlling cellular respiration [41]. Increasing evidence strongly supports that NO-mediated neurotoxicity, as seen in neurodegenerative diseases, is mediated by mitochondrial dysfunction [42,43,44]. Finally, we retrieved the Lsamp protein that mediates axon targeting and selective neuronal growth [34]. In particular, one GO biological process linked to this protein is ‘locomotory exploration behavior GO:0035641’. In this context, it has been shown that genetic deletion of Lsamp in mice causes exaggerated behavioral activation in novel environments, what the authors attribute to changes in neurotransmitter function and to alterations in synaptic connectivity [35]. More specifically, additional studies appoint altered function of 5-hydroxytryptamin, GABA-, and dopaminergic-systems in Lsamp-deficient mice. Moreover, an association was previously found between *LSAMP* gene polymorphisms and major depressive disorder, whereby patients presented with a significantly increased ratio between protective and risk *LSAMP* haplotypes compared to healthy volunteers [45].

Proteomic analysis of two-week (un)treated hippocampal tissue indicated synaptopathy featured by altered G-protein-coupled-receptor signaling and Golgi-lumen acidification. The finding of altered GPCR signaling can be explained by the upregulation of the Gabbr1 protein, i.e., γ-aminobutyric acid (GABA) B1, a subunit of the neuronal GABA B receptor. Neurons communicate with others to form network in the brain through the release and detection of neurotransmitters via ionotropic receptors, which mediate fast responses, and metabotropic receptors, which induce slow and long-term plasticity regulations [46]. One prominent metabotropic receptor subtype is the GABA B receptor, located both pre- and post-synaptically [47], which binds the main inhibitory neurotransmitter in the CNS, i.e., GABA. The GABA B receptor is a member of GPCR class C metabotropic glutamate receptors and functions as a heterodimer formed by the co-assembly of two subunits: GABA B1 and GABA B2 [48]. In this perspective, NO depletion by NOS inhibition was found to affect the GABA B receptor by the decreased release of GABA in the rat cerebral cortex [49]. Therefore, the increased expression of the GABA B1 subunit, the agonistic subunit of the GABA B heterodimer [33], might be an attempt to counterbalance the decreased GABA production and release. Normally, inhibitory GABAergic neurons maintain the inhibitory tone that counterbalances neuronal excitation in different brain regions through GABA release. When this balance is perturbed, neuronal hyperexcitation occurs, and seizures may ensue [50], as we observed in the mammalian phenotype analysis. Indeed, homeostatic changes in the expression of GABA B have been found to affect cognitive processing in neurodevelopmental disorders, i.e., autism spectrum disorders, fragile X syndrome, and Down’s syndrome, and neurodegenerative conditions, i.e., Alzheimer’s disease and epilepsy [51]. Additionally, a recent study performed an exome-wide rare variant analysis of six AD biomarkers (β-amyloid, total/phosphorylated tau, NfL, YKL-40, and Neurogranin) to discover genes associated with these markers. The authors found rare variants in the GABBR2 gene, which was identified with non-Alzheimer’s disease synaptic functioning, reinforcing the notion that short-term NOS inhibition affects synaptic functioning in non-pathological brain aging [52]. Furthermore, we found decreased expression of the Ube3a protein. GABAergic neuron-specific loss of Ube3a was previously shown to cause Angelman syndrome, a rare neurodevelopmental disorder characterized by abnormal electroencephalogram patterns and also enhanced seizure susceptibility, as we also observed in our mammalian phenotype analysis [53,54,55]. Studies in an Angelman syndrome mouse model (UBE3Am−/p+) showed deficits in experience-dependent synaptic plasticity [56] and synapse development [57]. Moreover, Ube3a is known to bind and ubiquitinate diverse substrates of the Golgi apparatus. In this perspective, studies in human shRNA transfected Ube3a knockdown cell lines, and UBE3Am−/p+ mouse neurons revealed elevated pH levels or acidification of the Golgi apparatus, aligning our findings, which were associated with a marked reduction in protein sialylation [58]. In the brain, protein sialylation is important for the regulation of synapse formation, neuronal excitability, neurite outgrowth, and neuron–glia interactions, among others [59]. Altogether we found GPCR-induced synaptopathy featured by hyperexcitability and acidification of the Golgi apparatus in 2-week L-NAME-treated hippocampi, which enhances the susceptibility of seizure development.

We were able to find one upregulated DEP in eight-week-treated LNAME hippocampi, namely, Tmsb10. Tmbs10 belongs to the beta-thymosin peptide family and plays an important role in the organization of the cytoskeleton. More specifically, it binds to and sequesters actin monomers and therefore inhibits actin polymerization [34]. Actin filaments together with microtubules and other cytoskeletal proteins orchestrate the structural organization of synapses and thus manage their formation, stability and plasticity. More specifically, synapses critically depend on the cytoskeleton for the transport and delivery of proteins from the nerve soma, for protein synthesis, as well as for surface diffusion of membrane proteins [60]. Continuing on the results of two-week L-NAME-treated hippocampi displaying neuronal hyperexcitability and an increased seizure susceptibility, acute depolymerization and long-term actin remodeling was previously shown in acute and chronic chemical mouse models of epilepsy [61,62], thus potentially contributing to epileptogenesis. Likewise, stabilization of actin polymerization with dexamethasone was recently shown to reduce the damage to actin filaments in pilocarpine-induced temporal lobe epilepsy [63], again highlighting the unbalance of actin cytoskeleton assembly and disassembly in the epileptic brain.

After sixteen weeks of NOS inhibition with L-NAME and the point reached of significantly deteriorated visuospatial learning and memory of treated C57BL/6 mice [21], 120 DEPs were found in their hippocampi. In accordance with our findings of 2-week L-NAME-treated hippocampi, we observed the downregulation of the Gabbr2, GABA B receptor subunit 2, alongside a downregulation of Sfn, 14-3-3 protein sigma. These proteins have been implicated in a variety of cellular processes, including G-protein-mediated signal transduction and regulation of synaptic transmission [64]. Furthermore, network analysis of the DEPs revealed the importance of Mapt as a hub protein being downregulated. Mapt, microtubule-associated protein tau, is a common pathological hallmark of tauopathy spectrum disorders, including AD, frontotemporal lobar degeneration, and Parkinson’s disease [65,66,67]. In this perspective, loss of NOS activity was shown to promote tau phosphorylation in mouse models of AD [68,69]. In addition, tau hyper-phosphorylation is a characterized pathology in the epileptic brain [70,71], and its pharmacological inhibition provided anti-seizure effects in several animal models of epilepsy [72,73,74]. Tau phosphorylation prompts the formation of insoluble full-length or truncated oligomeric tau species that participate in aggregation seeding and pathological propagation through the brain via a prion-like mechanism [75]. In addition, previous work showed that selective disruption of inhibitory synapses leads to neuronal hyperexcitability at an early stage of tauopathy in rTg4510 mice. More precisely, the authors found that tau induced the disruption of inhibitory synapses and hypothesized it to be a critical trigger of progressive neurodegeneration, resulting in massive neuronal loss [76]. While the authors designate tau to cause neuronal hyperexcitability, our proteomic analysis contradicts their finding and emphasizes the presence of neuronal excitability and seizure susceptibility prior to tauopathy upon treatment with a non-selective NOS inhibitor in C57BL/6 mice. Therefore, more precise research is needed to clarify the link between NO and tauopathy in sporadic and familial murine models of tauopathy. Another outcome of our analysis was ribosomal functioning. Pronounced ribosomal deficiencies appear in regions where tau pathology is evident [77]. Indeed, tau binds to ribosomes in the brain, and this interaction is enhanced in tauopathy brains, consequently reducing global protein synthesis in neurons [78]. Synaptic functionality depends on constant protein synthesis. Therefore, neurons are particularly vulnerable to a chronic reduction of RNA translation [79], which contributes to the pathogenesis of several neurodegenerative diseases, including tauopathies [80,81,82]. One peculiar finding was the downregulation of Tmsb10 after 16 weeks of L-NAME treatment, while this protein was significantly upregulated after 8 weeks of L-NAME treatment. In relation to its presence in the hippocampi after sixteen weeks of treatment, tau neurotoxicity correlates with the accumulation of filamentous actin and the formation of actin-rich rods in *Drosophila* and mouse models of tauopathy [83]. All this therefore suggests that tau phosphorylation and aggregation nullify the action and expression of Tmsb10, resulting in the accumulation and abnormal bundling of actin. Additionally, Mapt could be linked to the Rab10 hub protein, a key regulator of intracellular membrane trafficking, mediating the formation, transport, and fusion of vesicles with membranes. Since activation of Rab10 promotes axonal membrane trafficking, its downregulated expression might indicate the decreased vesicle numbers and axonal transport aligning our mouse mammalian phenotypic analysis.

Altogether, our findings implicate mitochondrial dysfunction, altered G-protein-coupled-receptor signaling, and the associated presence of seizures and altered emotional behavior after 2 weeks of L-NAME treatment, cerebral presence of seizure-related hyperexcitability after 8 weeks of treatment, followed by ribosomal dysfunction and tauopathy after 16 weeks of treatment. These pathological processes can be linked to the progressive molecular signatures underlying cognitive decline in C57BL/6 mice after L-NAME treatment and pinpoint potential targets of interest for tauopathy treatments. In addition, these results indicate the importance of NO not only in AS, but also in tauopathy and therefore potentially the mechanistic convergence between AS and cognitive decline.

Since L-NAME has a structure similar to L-arginine, and amino acid transporters are expressed at the blood–brain barrier [84], L-NAME is expected to cross the blood–brain barrier via arginine transporters. L-NAME has a 72% probability of crossing the blood–brain barrier [85], making it conceivable that cognitive decline following treatment with L-NAME is solely due to the inhibition of nNOS in the brain, independent of AS. However, because mice were treated with a relatively low dose of L-NAME in this study [22,23], and because L-NAME exhibits the lowest inhibitory constant (Ki) value for nNOS compared to eNOS and iNOS [86,87], it is likely that cerebral nNOS activity was inhibited. Limitations of this study are the fact that only male mice were used and the fact that only hippocampal and cortical brain tissues of one cerebral hemisphere were analyzed.

## 5. Conclusions

We conclude that the progressive effect of L-NAME treatment points towards mitochondrial and actin-related cytoskeletal dysfunction. Two weeks of L-NAME treatment implicates altered G-protein-coupled-receptor signaling in the nerve synapse and associated neuronal seizure-like hyperexcitability followed by actin polymerization after eight weeks and eventually neuronal ribosomal dysfunction and tauopathy after sixteen weeks in hippocampal tissue of C57BL/6 mice.

## Figures and Tables

**Table 1:** List of DEPs after factorial two-way ANOVA with Bonferroni–Hochberg correction for the interaction ‘Time “ Treatment”.

Uniprot ID	Protein ID	Protein Name	PAdj
P62073	Timm10	Mitochondrial import inner membrane translocase subunit Tim10	0.00328269
Q6ZWY8	Tmsb10	Thymosin beta-10	0.03230452
Q8BLK3	Lsamp	Limbic system-associated membrane protein	0.03230452

**Table 2:** List of DEPs between the 2-week (un)treated hippocampal protein datasets. Overall, proteins are ranked by *p*-value from most significant to least significant.

Uniprot ID	Protein ID	Protein Name	Log Fold Change	PAdj
O08759	Ube3a	Ubiquitin-protein ligase E3A	−3.67	0.0013
Q9WV18	Gabbr1	Gamma-aminobutyric acid type B receptor subunit 1	1.19	0.00919
E9QAM5	Helz2	Helicase with zinc finger domain 2	−2.11	0.00979
P53996	Cnbp	CCHC-type zinc finger nucleic acid binding protein	−3.19	0.0182
Q91WK2	Eif3h	Eukaryotic translation initiation factor 3 subunit H	4.62	0.0266
O35737	Hnrnph1	Heterogeneous nuclear ribonucleoprotein H	0.833	0.0266
P16858	Gapdh	Glyceraldehyde-3-phosphate dehydrogenase	0.961	0.0333
Q9DBL1	Acadsb	Short/branched chain specific acyl-CoA dehydrogenase, mitochondrial	0.916	0.0333
P60710	Actb	Actin, cytoplasmic 1	0.907	0.0394

**Table 3:** Top 10 most significant MGI Mammalian Phenotype terms for the DEP list of 2-week L-NAME-untreated and -treated hippocampal tissue of C57BL/6 mice. Terms are ranked by *p*-value from most significant to less significant.

	Name	Adjusted *p*-Value
1	absence seizures (MP:0003216)	0.001854
2	audiogenic seizures (MP:0001496)	0.001854
3	abnormal emotion/affect behavior (MP:0002572)	0.003941
4	abnormal liver size (MP:0004848)	0.02861
5	decreased prostate gland weight (MP:0004962)	0.02861
6	increased circulating prolactin level (MP:0005124)	0.02861
7	abnormal inhibitory postsynaptic potential (MP:0002911)	0.02861
8	decreased embryonic neuroepithelial cell proliferation (MP:0012706)	0.02861
9	abnormal cephalic neural fold morphology (MP:0011259)	0.02861
10	abnormal gas homeostasis (MP:0003948)	0.02861

**Table 4:** List of DEPs between the 8-week (un)treated hippocampal protein datasets.

Uniprot ID	Protein ID	Protein Name	Log Fold Change	PAdj
Q6ZWY8	Tmsb10	Thymosin beta-10	2.70	0.00294

**Table 5:** Top 10 most significant MGI Mammalian Phenotype terms for the DEP list of 16-week L-NAME-untreated and -treated hippocampal tissue of C57BL/6 mice. Terms are ranked by *p*-value from most significant to least significant.

	Name	PAdj
1	abnormal synaptic vesicle number MP:0004792	0.00006251
2	abnormal axon extension MP:0003651	0.0006462
3	decreased body weight MP:0001262	0.001084
4	abnormal miniature excitatory postsynaptic currents MP:0004753	0.005056
5	abnormal synapse morphology MP:0009538	0.005056
6	abnormal neurotransmitter level MP:0002204	0.005056
7	hyperactivity MP:0001399	0.01120
8	abnormal innervation MP:0002184	0.03494
9	abnormal barrel cortex morphology MP:0003989	0.04765
10	abnormal hippocampus physiology MP:0012006	0.04765

## Data Availability

Data are available via ProteomeXchange with identifier PXD033646. All data processing code is freely available on GitHub as open source under the Apache 2.0 license at https://github.com/adamscharlotte/L-NAME-Proteomic-Assessment (accessed on 18 March 2022).

## Appendix A

List of DEP proteins between the 16-week-(un)treated hippocampal protein datasets. Proteins are ranked by *p*-value from most to least significant.

**Uniprot ID**	**Protein ID**	**Protein Name**	**Log Fold Change**	**PAdj**
P10637	Mapt	Microtubule-associated protein tau	−0.481	0.000184
Q6IRU5	Cltb	Clathrin light chain B	−0.365	0.000381
Q3TFQ1	Spryd7	SPRY domain-containing protein 7	−1.94	0.000381
Q9Z1P6	Ndufa7	NADH dehydrogenase [ubiquinone] 1 alpha subcomplex subunit 7	−0.565	0.000381
Q61033	Tmpo	Lamina-associated polypeptide 2, isoforms beta/delta/epsilon/gamma	−1.29	0.00106
P33173	Kif1a	Kinesin-like protein KIF1A	−0.449	0.00259
Q8JZU2	Slc25a1	Tricarboxylate transport protein, mitochondrial	−1.51	0.00289
P63213	Gng2	Guanine nucleotide-binding protein G(I)/G(S)/G(O) subunit gamma-2	−0.482	0.00423
P56393	Cox7b	Cytochrome c oxidase subunit 7B, mitochondrial	−1.06	0.00423
P07356	Anxa2	Annexin A2	0.694	0.00429
Q8VDM4	Psmd2	26S proteasome non-ATPase regulatory subunit 2	−0.521	0.00439
P15327	Bpgm	Bisphosphoglycerate mutase	3.63	0.00439
Q60870	Reep5	Receptor expression-enhancing protein 5	−1.33	0.00537
Q6NS82	Retreg2	Reticulophagy regulator 2	−0.698	0.00642
P15209	Ntrk2	BDNF/NT-3 growth factors receptor	−0.552	0.00661
P22315	Fech	Ferrochelatase, mitochondrial	1.25	0.00661
Q8BFU3	Rnf214	RING finger protein 214	−1.28	0.00661
Q99MN9	Pccb	Propionyl-CoA carboxylase beta chain, mitochondrial	−0.526	0.00661
P04444	Hbb-bh1	Hemoglobin subunit beta-H1	−1.16	0.00671
Q61548	Snap91	Clathrin coat assembly protein AP180	−0.434	0.00671
Q62418	Dbnl	Drebrin-like protein	−0.436	0.00682
Q99LT0	Dpy30	Protein dpy-30 homolog	−0.565	0.00682
Q8R191	Syngr3	Synaptogyrin-3	−0.877	0.00722
P62242	Rps8	40S ribosomal protein S8	−0.506	0.00722
P55821	Stmn2	Stathmin-2	−0.829	0.00888
Q9ERE7	Mesd	LRP chaperone MESD	−0.627	0.00928
Q8BJU0	Sgta	Small glutamine-rich tetratricopeptide repeat-containing protein alpha	−0.53	0.00977
Q9JKV1	Adrm1	Proteasomal ubiquitin receptor ADRM1	−1.03	0.0102
Q922J3	Clip1	CAP-Gly domain-containing linker protein 1	−1.31	0.0123
P48678	Lmna	Prelamin-A/C	−0.507	0.0124
Q6ZQ58	Larp1	La-related protein 1	−1.45	0.013
P21279	Gnaq	Guanine nucleotide-binding protein G(q) subunit alpha	−0.43	0.013
Q8C5R8	Prps1l1	Ribose-phosphate diphosphokinase	−1.15	0.013
Q8VD37	Sgip1	SH3-containing GRB2-like protein 3-interacting protein 1	−0.41	0.013
Q8BSZ2	Ap3s2	AP-3 complex subunit sigma-2	−0.939	0.013
Q9D1J3	Sarnp	SAP domain-containing ribonucleoprotein	−0.921	0.013
Q8C854	Myef2	Myelin expression factor 2	−0.636	0.013
Q9WUM4	Coro1c	Coronin-1C	−0.383	0.0131
P62267	Rps23	40S ribosomal protein S23	−2.34	0.0138
P61089	Ube2n	Ubiquitin-conjugating enzyme E2 N	−0.461	0.0138
Q9QUR8	Sema7a	Semaphorin-7A	−1.96	0.0147
P11983	Tcp1	T-complex protein 1 subunit alpha	−0.319	0.0163
Q3V3R1	Mthfd1l	Monofunctional C1-tetrahydrofolate synthase, mitochondrial	−0.435	0.0163
P26645	Marcks	Myristoylated alanine-rich C-kinase substrate	−0.596	0.0165
Q9D883	U2af1	Splicing factor U2AF 26 kDa subunit	−0.851	0.0173
O88485	Dync1i1	Cytoplasmic dynein 1 intermediate chain 1	−0.675	0.0179
Q9CY58	Serbp1	Plasminogen activator inhibitor 1 RNA-binding protein	−0.785	0.0179
Q9Z2W9	Gria3	Glutamate receptor 3	−0.809	0.0179
P61027	Rab10	Ras-related protein Rab-10	−0.458	0.0179
Q8CCT4	Tceal5	Transcription elongation factor A protein-like 5	−0.761	0.0179
Q9JKS5	Habp4	Intracellular hyaluronan-binding protein 4	−2.23	0.0179
Q9Z2D6	Mecp2	Methyl-CpG-binding protein 2	−0.618	0.0179
Q8VHW2	Cacng8	Voltage-dependent calcium channel gamma-8 subunit	−1.85	0.0189
O89086	Rbm3	RNA-binding protein 3	−0.591	0.0191
P27661	H2ax	Histone H2AX	−0.859	0.02
Q9QYX7	Pclo	Protein piccolo	−0.591	0.0203
O09117	Sypl1	Synaptophysin-like protein 1	−0.807	0.0226
Q9DAK9	Phpt1	14 kDa phosphohistidine phosphatase	−0.747	0.023
Q91XV3	Basp1	Brain acid soluble protein 1	−0.591	0.0232
Q9Z2X1	Hnrnpf	Heterogeneous nuclear ribonucleoprotein F	−0.972	0.0232
Q9EPN1	Nbea	Neurobeachin	−1	0.0232
Q99KB8	Hagh	Hydroxyacylglutathione hydrolase, mitochondrial	−0.79	0.0232
Q8BLK3	Lsamp	Limbic system-associated membrane protein	−0.447	0.0232
Q91VR8	Brk1	Protein BRICK1	−0.636	0.0232
P11438	Lamp1	Lysosome-associated membrane glycoprotein 1	−2.77	0.0232
P63030	Mpc1	Mitochondrial pyruvate carrier 1	0.583	0.0232
Q8BGU5	Ccny	Cyclin-Y	−1.66	0.0232
Q8R5C5	Actr1b	Beta-centractin	−0.567	0.0232
Q9CXY6	Ilf2	Interleukin enhancer-binding factor 2	−0.553	0.0232
F8VQC1	Srp72	Signal recognition particle subunit SRP72	−1.29	0.0232
O88384	Vti1b	Vesicle transport through interaction with t-SNAREs homolog 1B	−1.05	0.0237
Q6PFD5	Dlgap3	Disks large-associated protein 3	−0.663	0.024
Q6ZWU9	Rps27	Ubiquitin-40S ribosomal protein S27a	−0.748	0.0245
F6ZDS4	Tpr	Nucleoprotein TPR	−0.979	0.0253
Q9WUM3	Coro1b	Coronin-1B	−0.43	0.0253
Q9CPT4	Mydgf	Myeloid-derived growth factor	−1.1	0.0255
Q8BGY7	Fam210a	Protein FAM210A	−0.831	0.0261
Q9CR00	Psmd9	26S proteasome non-ATPase regulatory subunit 9	−0.754	0.0261
Q9D7H3	RtcA	RNA 3′-terminal phosphate cyclase	−0.973	0.0261
P61294	Rab6b	Ras-related protein Rab-6B	−1.08	0.0267
Q9CZM2	Rpl15	60S ribosomal protein L15	−0.517	0.028
P62082	Rps7	40S ribosomal protein S7	−0.621	0.029
Q80T41	Gabbr2	Gamma-aminobutyric acid type B receptor subunit 2	−0.738	0.0298
Q6PH08	Erc2	ERC protein 2	−0.73	0.0298
P06837	Gap43	Neuromodulin	−0.683	0.0316
Q3UGC7	Eif3j1	Eukaryotic translation initiation factor 3 subunit J-A	−0.633	0.0316
P56382	Atp5f1e	ATP synthase subunit epsilon, mitochondrial	−0.57	0.0344
Q8VEK0	Tmem30a	Cell cycle control protein 50A	−0.599	0.0365
O55022	Pgrmc1	Membrane-associated progesterone receptor component 1	−0.401	0.0379
Q9CR51	Atp6v1g1	V-type proton ATPase subunit G 1	−0.707	0.0379
P61021	Rab5b	Ras-related protein Rab-5B	−0.71	0.0379
P48453	Ppp3cb	Serine/threonine-protein phosphatase 2B catalytic subunit beta isoform	−0.57	0.0379
Q8BIG7	Comtd1	Catechol O-methyltransferase domain-containing protein 1	−0.846	0.0392
Q80UP3	Dgkz	Diacylglycerol kinase zeta	−1.7	0.0393
Q8BSL7	Arf2	ADP-ribosylation factor-like protein 2-binding protein	−0.953	0.0393
P62073	Timm10	Mitochondrial import inner membrane translocase subunit Tim10	−0.789	0.0401
P83882	Rpl36a	60S ribosomal protein L36a	−1.05	0.0401
P70175	Dlg3	Disks large homolog 3	−0.62	0.0406
O88533	Ddc	Aromatic-L-amino-acid decarboxylase	−1.15	0.0431
Q5SVL6	Rap1gap2	Rap1 GTPase-activating protein 2	−1.14	0.0431
Q61792	Lasp1	LIM and SH3 domain protein 1	−0.772	0.0438
P61514	Rpl37a	60S ribosomal protein L37a	−1.52	0.0454
P28667	Marcksl1	MARCKS-related protein	−0.745	0.0454
Q3UHB8	Ccdc177	Coiled-coil domain-containing protein 177	−1.31	0.0454
Q8BTM8	Flna	Filamin-A	−0.876	0.0454
P13707	Gpd1	Glycerol-3-phosphate dehydrogenase [NAD(+)], cytoplasmic	−0.529	0.0454
Q6ZWY8	Tmsb10	Thymosin beta-10	−2.83	0.0454
Q61699	Hsph1	Heat shock protein 105 kDa	−0.333	0.0454
P62918	Rpl8	60S ribosomal protein L8	−0.457	0.0454
Q8BMG7	Rab3gap2	Rab3 GTPase-activating protein non-catalytic subunit	−1.59	0.0455
Q5XJY5	Arcn1	Coatomer subunit delta	−0.864	0.0455
Q9ERR1	Ndel1	Nuclear distribution protein nudE-like 1	−1.32	0.0463
Q9QZD9	Eif3i	Eukaryotic translation initiation factor 3 subunit I	−0.682	0.0465
O70492	Snx3	Sorting nexin-3	−0.575	0.0465
P61222	Abce1	ATP-binding cassette sub-family E member 1	−0.705	0.0478
O55091	Impact	Protein IMPACT	−0.575	0.0481
P15532	Nme1	Nucleoside diphosphate kinase A	−0.62	0.0481
O70456	Sfn	14-3-3 protein sigma	−1.02	0.0481
B0V2N1	Ptprs	Receptor-type tyrosine-protein phosphatase S	−0.667	0.0497
Q9WV55	Vapa	Vesicle-associated membrane protein-associated protein A	−0.631	0.0497
